# Effects of Educational Counseling as Solitary Therapy for Chronic Primary Tinnitus and Related Problems

**DOI:** 10.1155/2018/6032525

**Published:** 2018-06-26

**Authors:** Yu-Qing Liu, Zhi-Ji Chen, Gang Li, Dan Lai, Peng Liu, Yun Zheng

**Affiliations:** ^1^Hearing Center, Department of Otolaryngology-Head and Neck Surgery, West China Hospital of Sichuan University, Chengdu, China; ^2^Hearing Center, Guizhou Provincial People's Hospital, Guiyang, China; ^3^Department of Otolaryngology-Head and Neck Surgery, Southwest Medical University, Luzhou, China; ^4^Department of Otolaryngology-Head and Neck Surgery, The First Affiliated Hospital of Guangzhou University of TCM, Guangzhou, China

## Abstract

The aim of this study was to evaluate the early and sustained effects of tinnitus educational counseling on chronic primary tinnitus and related problems. A descriptive longitudinal cohort study was conducted with 159 adult patients suffering from chronic primary tinnitus and sleep problems. All patients received tinnitus educational counseling, sleep adjustment, and vegan dietary advice. At short-term assessment within 3 months and long-term follow-up at 6–26 months, perceived changes in tinnitus were assessed with the Tinnitus Handicap Inventory (THI) and the Tinnitus Evaluation Questionnaire (TEQ), respectively. In TEQ, the volume of subjective tinnitus was scored according to realistic environments in which tinnitus could be heard. Sleep quality was assessed with questionnaires developed in our laboratory. Most of the subjects showed significant early improvement in their THI scores (96/159, 60.38%; from 46.11 ± 22.74 to 31.94 ± 20.41,* t* = 11.16,* p* < 0.001, Cohen's* d* = 0.66). Tinnitus volume (39/159, 24.53%, from 2,2 to 2,1,* z* = -3.56,* p* < 0.001) and sleep quality (68/159, 42.77%; from 7.13 ± 3.11 to 6.31 ± 2.75,* t* = 3.73,* p* < 0.001, Cohen's* d* = 0.28) were also improved. Long-term follow-up TEQ results indicated that tinnitus loudness, the impact of tinnitus on sleep, concentration, and emotional state were all improved since the prior consultation (*p* = 0.001, 0.026, 0.012, and <0.001). Short-term improvement of tinnitus severity correlated directly with improvement of sleep quality (odds ratio (OR) = 0.30, 95% confidence interval (CI): 0.14–0.64,* p* = 0.002), initial THI score (OR = 1.02, 95% CI: 1.01 to 1.04,* p* = 0.006), compliance with sleep advice (OR = 2.27, 95% CI: 1.02–5.05,* p* = 0.044), and nervous disposition (OR = 2.80, 95% CI: 1.25–6.30,* p* = 0.013). A future randomized controlled trial would be carried out to examine the effect of sole tinnitus educational counseling.

## 1. Introduction

Tinnitus is the persistent and bothersome perception of sound in the absence of an external source. The prevalence of tinnitus lasting for more than 5 min at a time is reported to range from 11.9% to 30.3% [1]. Traditional approaches to the treatment of tinnitus are largely ineffective [2]. Tinnitus educational counseling is a form of psychological therapy that is cost-effective [3], even in the long term [4]. Such counseling, which includes the provision of general information about the physiology of hearing, pathophysiology of tinnitus, audiometric testing, and strategies for the minimization and control of tinnitus, has been found to be as effective as tinnitus masking and tinnitus retraining therapy within periods as short as 6 months [4]. Patients have also included counseling and education, as well as cognitive behavioral therapy (CBT), among tinnitus therapy methods that they have found to be most effective [5].

The provision of advice regarding sleep and lifestyle modifications is another form of psychological therapy for tinnitus [6], as even relatively healthy people with chronic tinnitus may suffer from sleep-related problems [7]. Sleep disturbances increase the risk of tinnitus [8] and associated functional impairment [9, 10]. Insomnia increases patients' negative reactions to tinnitus [7, 11], leading to depression [12]. The association between functional impairment and sleep disturbance becomes more noticeable over time [13]. The evaluation and treatment of sleep disturbances help reduce patients' subjective handling of tinnitus [11] and are important components of tinnitus management, resulting in improved outcomes [10, 14]. The role of diet has also been mentioned in some studies [15, 16].

However, studies regarding the impact of sleep on tinnitus management are relatively scarce compared with those examining the control of other subjective symptoms, such as pain [17–19]. Tinnitus and pain have similar characteristics, such as their subjective nature, the limited success of interventions, and being characterized by hypersensitivity to sensory stimulation [20]. We analyzed the impact of sleep adjustment and sleep quality on tinnitus severity. We also examined the potential influence of personality characteristics on tinnitus [21]. Tinnitus educational counseling and sleep adjustment in tinnitus sufferers were included in this study. Early and long-term (>6 months) effects of tinnitus educational counseling were monitored together with the influence of personality characteristics on these effects.

## 2. Materials and Methods

### 2.1. Subjects

The demographic and clinical details of 159 adult (age > 18 years) patients who attended special clinics for tinnitus, hearing loss, and vertigo at the Hearing Center, Department of Otolaryngology-Head and Neck Surgery, West China Hospital of Sichuan University, between January 2015 and February 2017 were analyzed prospectively. Our institutional review board had no ethical concerns and approved this study, which was implemented in accordance with the board's medical-ethical standards. Subjects included in the study population provided consent for the use of their clinically obtained data.

The criteria for patient selection were the following: (a) primary complaint of tinnitus lasting more than 6 months, with or without sensorineural hearing loss [3]; (b) clinical evaluation including medical history, physical examination, and comprehensive audiological examination; (c) availability of complete evaluation records and lifestyle questionnaires; (d) unhealthy sleep habits, such as going to bed late and rising late and/or insufficient or excessive duration of sleep, and dissatisfaction with sleep quality; and (e) receipt of oral and written sleep and lifestyle advice in addition to one-on-one tinnitus educational counseling.

### 2.2. Intervention

A combination of tinnitus educational counseling and sleep/lifestyle advice was delivered by a trained audiology technician and then by the treating physician during the first consultation. Detailed counseling by the trained audiology technician (1–2 h) and the physician (10–20 min) was ensured for all subjects. Tinnitus educational counseling consisted of three aspects: (a) explanation of the physiology of hearing, pathophysiology of tinnitus, audiometric testing, and strategies for the minimization and control of tinnitus; (b) discussion of the relationship between tinnitus and sleep and the importance of sleep hygiene; and (c) reassurance that “tinnitus is like a well-meaning alarm” and thus a useful health reminder. The concept of sleep hygiene includes the proper timing and sufficient duration of, and subjective satisfaction with, sleep, translating into highly effective sleep and enhanced alertness during daily activities [22]. In the* Yellow Emperor's Canon* [23], a classic traditional Chinese medicine text, recommendations for healthful sleep include falling asleep by 11 pm and rising between 5 am and 7 am, thereby ensuring an in-bed sleeping window between 10 pm and 6 am. In consideration of daytime sleepiness and nonadaptation in some patients, a short nap before 2 pm for a maximum of 30 min was advised.

### 2.3. Data Collection

**Figure 1:**
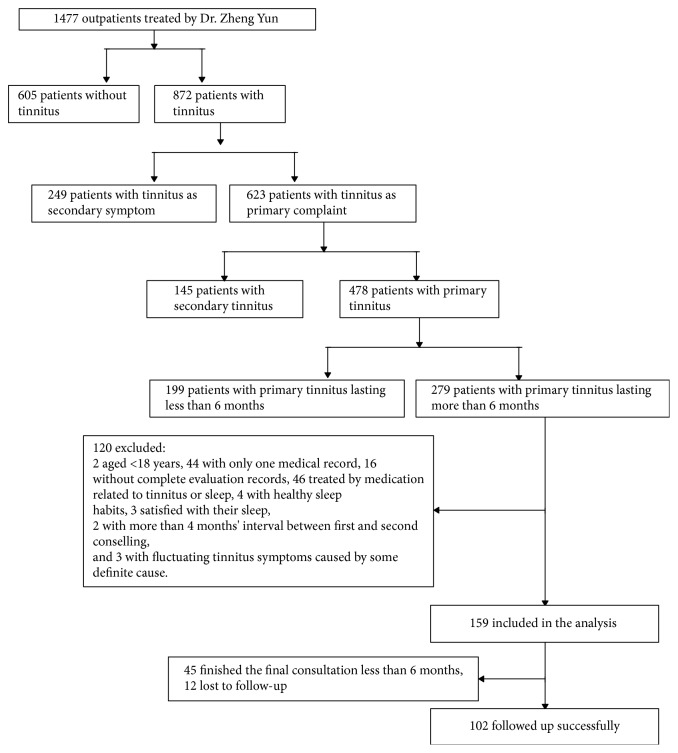
Flowchart of patient enrollment into the study.

The inclusion of cases into this study is summarized in the flowchart shown in Figure 1. Patients provided informed consent for the use of their deidentified data for education and research. All patients provided their basic personal information, including gender, age, level of education, occupation, marital status, living habits, contact information/address, and nationality. They also provided self-reported personality trait information, underwent audiometry, and assessments of tinnitus and sleep. Hearing assessed with a pure tone hearing threshold test at frequencies in the range of 250–8000 Hz demonstrated that all subjects had thresholds <25 dB hearing level, indicating normal hearing.

At each consultation, patients received 1–2 hours of tinnitus education, sleep hygiene information, and vegan dietary advice (i.e., not to eat any animal products and how to ensure sufficient nutrition from other staple foods). Their self-reported tinnitus status and lifestyle questionnaire responses were recorded. Tinnitus severity, tinnitus volume, and sleep quality were assessed during the first and subsequent consultations (mean interassessment interval, 30.70 ± 19.95 days). Initial and postintervention sleep qualities were described for the periods of 1–3 months before the intervention and 1–4 weeks before the second to last evaluation. The short- and long-term severity of tinnitus, in terms of the extent of impairment caused by the presence of tinnitus, were assessed with the Chinese-Mandarin versions of the Tinnitus Handicap Inventory (THI-CM) [24] and Tinnitus Evaluation Questionnaire (TEQ) [25], respectively, to assess the short-term and long-term changes.

The THI-CM consists of 25 items grouped into functional, emotional, and catastrophic subscales. Its test-retest reliability (Pearson's* r* = 0.98) and internal validity (Cronbach's *α* = 0.93) have been documented [24]. Total THI scores were taken to indicate five grades of tinnitus severity: Grade I (score, 0–16), slight tinnitus that is perceived only in a quiet environment and generally benign; Grade II (score, 18–36), mild tinnitus that can be masked easily and interferes with sleep only occasionally but not daily activities; Grade III (score, 38–56), moderate tinnitus that is pervasive but interferes with sleep or daily activities only occasionally; Grade IV (score, 58–76), severe tinnitus that is perceived most of the time and disturbs sleep patterns and daily activities often; and Grade V (score, 78–100), catastrophic tinnitus symptoms that disturb sleep and daily activities consistently [26]. Although a difference ≥ 20 points between pre- and postintervention scores is considered statistically significant [27], a difference of 7 points is accepted as clinically significant [28]. In this study, score changes of ≥7 points were taken to indicate a moderate change, and changes ≥ 20 points were taken to denote strong improvement or serious worsening. Changes of ≤6 points were recorded as “no change.” Subjects with THI-CM score reductions of ≥7 points were designated as the effective group, and all others formed the noneffective group.

The TEQ consists of six questions. The first five were the following: when is your tinnitus heard? (responses: no tinnitus, in quiet surroundings, in normal surroundings, and in noisy surroundings); is your tinnitus continuous or intermittent? (responses: no tinnitus, more absence than presence, absence is equal to presence, and less absence than presence); does your tinnitus affect your falling asleep? (responses: never, sometimes, often, and always); does your tinnitus affect your concentration? (responses: never, sometimes, often, and always); and does your tinnitus affect your emotions? (responses: never, sometimes, often, and always). The four possible answers to each of these questions correspond to a 0–3-point scale. The sixth item was a tinnitus severity score of tinnitus assessed by evaluator. The first five questions were used for follow-up evaluation and contrasted with the previous face-to-face assessments. Tinnitus volume was assessed by a combination of psychoacoustic methods [29] and the administration of a visual analogue scale (VAS) [30] and numeric rating scales [31]. The TEQ response to the first item, which reflects broadly accessible real-life situations, was used to evaluate tinnitus loudness.

We developed a five-item sleep quality survey that included the following items: difficulty falling asleep (No. 1), frequency of awakening after sleep onset (No. 2), difficulty returning to sleep after awakening in night sleep (No. 3), daytime sleepiness (No. 4), and number of times of awakening (No. 5). The response options for items 1–4 were “never,” “≤1–2 times per week,” “3–4 times per week,” and “≥5 times per week.” Response options for item No. 5 were “never,” “1 time per night,” “2 times per night,” and “≥3 times per night.” Scores were assigned to these responses in ascending order. The criterion for insomnia was at least three times per week of sleep initiation or maintenance problems [32]. According to the principle of change in health-related quality of life [33], a change of more than half a standard deviation at baseline was taken to indicate improvement or worsening of sleep quality.

To assess the participants' personality characteristics, we developed a brief questionnaire with the following yes/no questions: Are you always impatient? Are you constantly anxious for others? Are you continually irritable? Are you nervous all the time? Are you frequently suspicious? Are you a perfectionist?

### 2.4. Follow-up Assessment

We contacted by phone the patients who completed counseling within the one-week unified study period. The follow-up period after the last counseling session ranged from 6 to 26 months (16.04 ± 4.56 months). Follow-up by telephone was considered to be more convenient than a clinic visit, e-mail, or online program. The TEQ questions were asked in the phone follow-up. In addition, we asked the following three questions: did you accept any other treatment apart from tinnitus educational counseling? (responses: no, yes); did you follow the advice on sleep adjustment? (responses: no, partly, or absolutely); and did you follow the vegan dietary advice provided? (responses: no, partly, or absolutely).

### 2.5. Statistical Analysis

Statistical analyses were performed with IBM SPSS Statistics 20, with a significance criterion of* p* < 0.05 considered to be statistically significant. Data were denoted as means with standard deviations or medians with interquartile ranges (IQRs). The mean total THI scores at the first and last consultations were compared using the paired-samples* t*-test. Scores for tinnitus volume and sleep quality were compared using the Wilcoxon signed-rank test. Factors related to improvement in tinnitus severity were determined by logistic regression analysis. The effect size (Cohen's* d*) was calculated according to the formula described previously [34]. The postintervention mean THI scores were deducted from the preintervention mean, and the result was divided by the pooled pre-/postintervention standard deviation. Stepwise logistic regression analysis was used to predict what factors were associated with the change in THI. Firstly, the demographic and clinical characteristics were compared between the effective group and noneffective group in short-term observation. Then, the characteristics with statistical differences in the two groups would be included in the regression analysis to predict the related factors.

## 3. Results

### 3.1. Subject Characteristics

This study included 159 patients (77 males and 82 females) with an average age of 44.86 ± 13.44 years. The mean of the pure-tone averages at 0.5 kHz, 1 kHz, 2 kHz, and 4 kHz was 29 dBHL. According to THI scores, 12 (7.55%) patients had Grade I, 50 (31.44%) had Grade II, 48 (30.19%) had Grade III, 29 (18.24%) had Grade IV, and 20 (12.58%) patients had Grade V tinnitus. Tinnitus was bilateral in 56 cases and unilateral in 103 cases (left, 54 cases; right, 49 cases). Nearly half (49.06%, 78/159) of the subjects met the criteria for insomnia, whereas 50.94% (81/159) did not have a serious sleep problem. Of 159 patients, 115 complied strictly with the sleep advice, going to bed before 10 pm and waking before 6 am, up to the time of the short-term assessment; they were classified as subjects who adjusted (group A). The remaining 44 patients did not change their usual sleep habits, which included going to bed after 11 pm and having <6 h sleep or waking after 8 am and having >8 h sleep; they were classified as subjects who did not adjust (group NA).

In total, 114 patients finished the final consultation at ≥6 months. Among them, 102 patients were followed up successfully within the same week (April 22–28, 2017). Thus, 18 patients completed 6–11 months' counseling, 25 patients completed 12–17 months, 30 patients completed 18–23 months, and 29 patients completed 24–26 months' counseling. Twelve patients were lost to follow-up, as they could not be contacted by telephone. The follow-up rate was thus 89.47%.

### 3.2. Overall Improvement of Tinnitus and Sleep Quality in the Short-Term (30.70 ± 19.95 Days) Assessment Period

Mean THI, tinnitus volume, and sleep quality scores declined from the first to the last assessment in 159 subjects. The mean THI score decreased from 46.11 ± 22.74 to 31.94 ± 20.41 (*t* = 11.16,* p* < 0.001). The postintervention effect size relative to preintervention one was moderate (Cohen's* d* = 0.66). THI score changes indicated that tinnitus became less severe in more than half (96/159, 60.38%) of the subjects. However, THI scores showed moderate worsening in 7 patients and serious worsening in 3 patients.

The postintervention tinnitus volume score (median, IQR: 2, 1) was also lower than that at baseline (median, IQR: 2, 2; *z* = −3.56, *p* < 0.001). Self-assessed tinnitus volume was mitigated in 39 (24.53%, 39/159) cases but worsened in 4 cases.

Sleep quality also improved, as shown by a significant reduction of the score from 7.13 ± 3.11 to 6.31 ± 2.75 (*t* = 3.73,* p* < 0.001). The effect size was small (Cohen's* d* = 0.28). Sleep quality improved in nearly half (42.77%, 68/159) of the subjects. The number of patients meeting the criterion for insomnia declined from 78 at baseline to 64 after intervention. Improvements in difficulty falling asleep, difficulty returning to sleep after awakening, and daytime sleepiness in patients who undertook sleep adjustment are shown in Table 1.

### 3.3. Factors Related to the Improvement of Tinnitus Severity in the Short- Term (30.70 ± 19.95 Days) Assessment Period

The demographic characteristics, initial THI scores, sleep quality at last contact, improvement in sleep quality, compliance with sleep adjustment, and nervous disposition differed significantly between effective group and noneffective group (*p* < 0.05) (Table 2). These five factors were treated as variables in a stepwise logistic regression analysis with forward selection intended to predict which factors were related to the change in THI. Logistic regression analysis indicated that tinnitus improvement correlated positively with improved sleep quality (OR = 0.30, 95% CI: 0.14–0.64,* p* = 0.002), initial THI score (OR = 1.02, 95% CI: 1.01–1.04,* p* = 0.006), compliance with sleep advice (OR = 2.27, 95% CI: 1.02–5.05,* p* = 0.044), and nervous disposition (OR = 2.80, 95% CI: 1.25–6.30,* p* = 0.013). Sleep quality determined at last assessment was removed from the model during the process of stepwise regression (*p *= 0.067).

### 3.4. Long-Term (16.04 ± 4.56 Months) Change of Tinnitus Indicated by TEQ

Among the 102 patients who received tinnitus counseling (6–26 months), 6 patients were prescribed short rounds of pharmacotherapy by doctors outside the hospital. Tinnitus and its impacts are described in Table 3. Seventy-eight patients reported that tinnitus no longer affected their falling asleep, 96 patients reported that tinnitus had stopped interrupting their concentration, and 64 patients reported that tinnitus no longer influenced their emotions. Tinnitus disappeared in 5 patients (Table 4). In addition, most patients continued to heed the sleep advice, following sleep timetables and adjusting their sleep, after the last consultation. 42 patients continued to go to bed and get up early (including 9 patients who did not change their previous sleep habits), and 38 patients followed either the early bedtime or early awakening routines. 22 patients did not change their late bedtime and awakening patterns.

## 4. Discussion

The intention of this study was to explore the short-term effects of tinnitus educational counseling as solitary therapy for chronic primary tinnitus and its long-term sustainability, taking into account related factors such as sleep adjustment and sleep quality improvement. The results suggest that tinnitus educational counseling is sufficient to satisfy many patients, as corroborated by previous studies [4]. The early and long-term assessments showed decreased severity of tinnitus and sustainment of the improvement. Personality characteristics, adherence to sleep hygiene, and improvement in sleep quality were related to the improvement of tinnitus severity.

THI scores improved significantly within 3 months in the majority of subjects, although the effect size was moderate. Tinnitus volume was also reduced significantly in nearly a quarter of the subjects. This study differed from other studies [4] with respect to the features of tinnitus educational counseling, which accounts for the better outcomes observed. In addition to conventional tinnitus education, we commented that tinnitus was a well-meaning alarm to remind individuals to attach more importance to health; study participants responded positively, similar to acceptance therapy [35], a psychotherapeutic approach that lessens emotional avoidance and increases the capacity for behavioral change [36]. Acceptance was strongly and inversely related to tinnitus severity causing impairment and attendant anxiety and depression [35]. Another characteristic of the treatment was adherence to sleep hygiene. Clear correlations were observed between sleep problems and erratic sleep hygiene [37]. Thus, a focused approach to participants' sleep schedules was beneficial, as it seemed to enhance the effect of tinnitus educational counseling. The results suggest that outcomes can be improved by a focus on sleep hygiene as a specific intervention in patients with tinnitus and sleep problems. However, the reasons for serious worsening of tinnitus and increased tinnitus volume in three cases each are not clear. The chance for improvement might have been dissipated by the rigidity of the approach [38]. Strict compliance with the specified sleep timing and vegan diet might have resulted in major changes in patients' lifestyles, and maladaptation, frustration, and anxiety might have resulted in worsening of tinnitus. A graduated and personalized approach may thus be more acceptable and yield better outcomes.

The sleep quality of patients with insomnia has been shown to be improved with sleep restriction [39], which involves implementation of a prescribed amount of total time allowed in bed. Subjective sleep quality, indicated by sleep onset latency, awakening time after sleep onset, and sleep efficiency, improved after sleep restriction, and the effect size was moderate to large [39]. Our results showed that sleep quality improved after tinnitus educational counseling in patients with tinnitus-related sleep problems through adherence to sleep hygiene, especially with regard to difficulty falling asleep, going back to sleep again after nocturnal awakening, and daytime sleepiness. Advice on sleep adjustment emphasized the timing of going to bed and awakening, in addition to sleep duration, rooted in the principle of health preservation in traditional Chinese medicine. This form of sleep adjustment was clearly beneficial, improving nocturnal sleep quality and daytime function. These results highlight the easier aspects of sleep improvement by personal adjustments, which were stressed properly during consultations. Nervous individuals seemed to experience greater benefits, suggesting that personality characteristics play influential roles; for example, neuroticism increases and extraversion decreases in patients with subjective idiopathic tinnitus [21]. As patients who are neurotic and introverted may take better advantage of psychiatric consultation, tinnitus education as solitary therapy could be more effective for them. In our daily clinic work, we have observed that some nervous patients were more likely to seek information about their tinnitus online and to be anxious about serious diseases related to it. Counseling could thus alleviate the negative effects of tinnitus. Therefore, personalized and targeted counseling with consideration of personality characteristics is advisable.

In a prospective behavioral therapy study with a 15-year follow-up period, tinnitus disappeared in 13 of 244 (5.3%) patients with chronic tinnitus [40]. In our 6–26-month follow-up of 102 patients by telephone, tinnitus had disappeared within 16 months after the initial contact in 5 (4.81%) patients, a resolution rate similar to that reported in the aforementioned 15-year follow-up study. Prediction of the time point at which tinnitus disappears completely would be difficult. However, these five patients had some common features, such as lesser tinnitus severity (Grades I–III) and occurrence of tinnitus only under quiet conditions (tinnitus could be heard in noisy surroundings in one case). The insistence on early sleep and awakening times also probably contributed to the resolution of tinnitus in these patients because of better motivation and effort on their parts.

The findings of this study suggest that the combination of tinnitus educational counseling and provision of sleep hygiene advice is a feasible management option for tinnitus in primary care, especially in underdeveloped areas. Although CBT or a combination of sound therapy and CBT-based counseling is recommended for tinnitus management [3, 41], these methods may be limited by the scarcity of clinical and trainee psychologists. To overcome this limitation, a simplified version of psychological intervention which can be implemented by other clinical professionals should be developed to enable the holistic treatment of tinnitus [42]. In this study, tinnitus educational counseling and advice on sleep hygiene were delivered primarily by a trainee audiology technician, and this approach may be an alternative for patients in regions without professional psychology services. General practitioners (GPs) are often the first professionals encountered by patients seeking help with their tinnitus, and most otolaryngologists see referred patients, typically from GPs [2]. The addition of therapy such as CBT or sound therapy might lead to greater improvement, and the number of cured patients might increase with longer periods of observation. The combination of tinnitus educational counseling and sleep hygiene, with an emphasis on self-management of sleep problems, is applicable in underdeveloped areas and in primary health care, and may be more cost-effective than sole tinnitus educational counseling for patients with chronic primary tinnitus, especially when accompanied by sleep problems.

### 4.1. Limitations

Because this study was descriptive and not a randomized controlled trial, it has some inherent limitations. Because the numbers of subjects who underwent sleep adjustment, hearing, and tinnitus assessments were not distributed equally, this work may be considered a pilot study. The timing and duration of follow-up were not consistent across the study sample due to differences in registration dates. In addition, we used a developed-in-house personality, sleep quality, and tinnitus volume survey instead of validated instruments (although the THI is validated). Confounding factors, including those related to lifestyle, diet, and exercise, could not be excluded. Baseline THI scores were <7 in three cases, preventing accurate assessment of the improvement of tinnitus severity.

## 5. Conclusion

The study results demonstrated that, by tinnitus education counseling, tinnitus severity of THI decreased in short-term observation, and the tinnitus volume and its negative impact on mood, sleep, and concentration had also improved in long-term follow-up. Changes in unhealthy sleep habits can reduce tinnitus severity and improve sleep quality. Patients with less severe tinnitus and healthy sleep patterns tend to be cured more easily. Thus, tinnitus education should not only include sleep hygiene but also emphasize patient compliance. Patient personality is an important factor, and nervous patients are more likely to benefit from educational counseling.

## Figures and Tables

**Table 1:** Pre- and postintervention sleep quality scores (*N* = 159).

**Subjects**	**Evaluation relative to intervention**	**Difficulty falling asleep, median (IQR)**	**Frequency of waking after sleep onset, median (IQR)**	**No. of times waking after sleep onset, median (IQR)**	**Difficulty returning to sleep after waking at midnight, median (IQR)**	**Daytime sleepiness, median (IQR)**	**Total,** **mean ± SD**
All	Pre	1 (1,2)	1 (1,2)	1 (1,2)	1 (1,2)	1 (1,1)	7.12 ± 3.07
	Post	1 (1,2)	1 (1,2)	1 (1,2)	1 (0,2)	1 (0,1)	6.31 ± 2.75
	Z	-2.341	-0.024	-1.688	-3.068	-4.093	-3.545
	p	0.019	0.981	0.091	0.002	<0.001	<0.001
A	Pre	2 (1,2)	1 (1,2)	2 (1,2)	2 (1,2)	1 (1,2)	7.39 ± 3.06
	Post	1 (1,2)	1 (1,2)	1 (1,2)	1 (0,2)	1 (1,1)	6.38 ± 2.79
	Z	-2.17	-0.39	-1.59	-2.87	-4.024	-3.525
	p	0.030	0.692	0.113	0.004	<0.001	<0.001
NA	Pre	1 (1,2)	1 (1,2)	1 (1,2)	1 (1,2)	1 (1,1)	6.41 ± 3.03
	Post	1 (1,2)	1 (1,2)	1(1,2)	1 (0,2)	1 (0,1)	6.11 ± 2.68
	Z	-0.938	-0.593	-0.632	-1.143	-1.091	-0.897
	p	0.348	0.553	0.527	0.253	0.275	0.370

A, with sleep adjustment; NA, without adjustment.

**Table 2:** Demographic and clinical characteristics of the effective and noneffective groups, defined by THI score.

**Characteristic**	**Effective** **(N = 96)**	**Noneffective** **(N = 63)**	***P***
Gender, no. of males : no. of females	50 : 46	27 : 36	0.255_ _^a^
Mean age ± SD, years	44.03 ± 13.12	46.13 ± 13.93	0.338_ _^b^
Hearing loss, N (%)	64 (66.7%)	46 (73.0%)	0.396_ _^a^
*Education degree, N (%)*			0.197
Primary school	7 (7.3%)	7 (11.1%)	
Junior high school	24 (25.0%)	10 (15.9%)	
Senior high school	16 (16.7%)	18 (28.6%)	
University	44 (45.8%)	27 (42.9%)	
Master's degree or higher	5 (5.2%)	1 (1.6%)	
*Character traits *			
Impatient	49 (51.0%)	41 (65.1%)	0.081_ _^a^
Anxious for others	43 (44.8%)	29 (46.0%)	0.878_ _^a^
Irritable	38 (39.6%)	17 (27.0%)	0.102_ _^a^
Nervous	42 (43.8%)	12 (19.0%)	0.001_ _^a^
Suspicious	11 (11.5%)	4 (6.3%)	0.281_ _^a^
Perfectionist	17 (17.7%)	17 (27.0%)	0.163_ _^a^

Median tinnitus duration, months (IQR)	24 (62)	24 (48)	0.798_ _^c^
Mean initial THI score ± SD	51.25 ± 20.85	38.60 ± 23.15	<0.001_ _^b^
Mean first-to-last-contact interval ± SD, days	35.41 ± 20.86	35.10 ± 20.14	0.926_ _^b^
Sleep quality at first contact	7.38 ± 2.98	6.76 ± 3.29	0.225_ _^b^
Sleep quality at last contact	5.85 ± 2.45	7.00 ± 3.05	0.010_ _^c^
Change in sleep quality, *N* (%)			
Improved	53 (55.2%)	15 (23.8%)	<0.001_ _^a^
Not improved	43 (44.8%)	48 (76.2%)	
Sleep advice compliance, *N* (%)			0.017_ _^a^
Better	76 (79.2%)	39 (61.9%)	
Worse	20 (20.8%)	24 (38.1%)	

THI, Tinnitus Handicap Inventory.

^a^Chi-squared test, ^b^one-way ANOVA, and ^c^Mann–Whitney *U* test.

**Table 3:** Short- and long-term changes in tinnitus indicated by TEQ (*N* = 102).

**Item**	**Time point**	**Tinnitus characteristic**				***P***
		**None**	**Quiet conditions**	**Normal conditions**	**Noisy conditions**	
Tinnitus loudness	First contact	0	29	42	31	0.098_ _^a^
	Last contact	0	40	43	19	
	Follow-up	5	60	22	15	0.001_ _^b^
		**Interval > duration**	**Interval ** ≈** duration**	**Duration > interval**	**Continuity**	

Tinnitus continuance	First contact	0	2	4	96	0.072_ _^a^
	Last contact	0	7	9	86	
	Follow-up	5	9	8	80	0.137_ _^b^
		**None**	**Sometimes**	**Often**	**Always**	

Impact on sleep	First contact	37	21	23	21	0.001_ _^a^
	Last contact	58	27	8	9	
	Follow-up	78	14	6	4	0.026_ _^b^

Impact on concentration	First contact	59	30	12	1	0.005_ _^a^
	Last contact	82	15	5	0	
	Follow-up	96	5	1	0	0.012_ _^b^

Impact on emotion	First contact	4	58	33	7	<0.001_ _^a^
	Last contact	17	70	14	1	
	Follow-up	64	35	2	1	<0.001_ _^b^

^a^First contact versus last contact; ^b^last contact versus follow-up.

**Table 4:** Characteristics of 5 patients with complete tinnitus resolution.

**Characteristic**	**Case ** **1**	**Case ** **2**	**Case ** **3**	**Case ** **4**	**Case ** **5**
Gender	Female	Female	Female	Male	Female
Age, years	44	65	51	24	48
Tinnitus side	Right	Bilateral	Right	Left	Bilateral
Hearing	Normal	Impaired	Normal	Normal	Impaired
Initial THI severity grade	I	II	II	III	II
Tinnitus loudness	In quiet	In noise	In quiet	In quiet	In quiet

*Durations, months*					
Tinnitus before first contact	6	24	13	11	30
Tinnitus cessation after initial education	2.7	15.5	6	11	7
Total tinnitus before cessation	11	39.5	19	22	37
Follow-up duration after initial education	16.7	22.5	22	12	12.5
Strict sleep adjustment	Until date	Until date	Until date	Until date	Until date
Diet adjustment	Controlled partly until date	Controlled partly until date	Controlled strictly before tinnitus cessation	Controlled strictly for initial 6 months	Controlled strictly for initial 11 months

## Data Availability

All data generated or analyzed during this study are included within the article.
